# Intra- and Inter-subject Variability in EEG-Based Sensorimotor Brain Computer Interface: A Review

**DOI:** 10.3389/fncom.2019.00087

**Published:** 2020-01-21

**Authors:** Simanto Saha, Mathias Baumert

**Affiliations:** School of Electrical and Electronic Engineering, The University of Adelaide, Adelaide, SA, Australia

**Keywords:** electroencephalography, brain computer interface, sensorimotor rhythms, transfer learning, inter-subject associativity

## Abstract

Brain computer interfaces (BCI) for the rehabilitation of motor impairments exploit sensorimotor rhythms (SMR) in the electroencephalogram (EEG). However, the neurophysiological processes underpinning the SMR often vary over time and across subjects. Inherent intra- and inter-subject variability causes covariate shift in data distributions that impede the transferability of model parameters amongst sessions/subjects. Transfer learning includes machine learning-based methods to compensate for inter-subject and inter-session (intra-subject) variability manifested in EEG-derived feature distributions as a covariate shift for BCI. Besides transfer learning approaches, recent studies have explored psychological and neurophysiological predictors as well as inter-subject associativity assessment, which may augment transfer learning in EEG-based BCI. Here, we highlight the importance of measuring inter-session/subject performance predictors for generalized BCI frameworks for both normal and motor-impaired people, reducing the necessity for tedious and annoying calibration sessions and BCI training.

## 1. Introduction

Brain computer interfaces (BCI) exploiting sensorimotor rhythms (SMR) have shown promise for both the improvement of motor performance in normal subjects and the rehabilitation of motor function in patients (Dobkin, 2007; Wang and Jung, 2011). The SMR can be elicited by motor imagery (MI) that shares common neurophysiological mechanisms with overt motor execution (ME), the former being more convenient for BCI users who cannot perform an overt ME task due to some degree of motor disability (Jeannerod, 1995; Lotze and Halsband, 2006; Zich et al., 2015; Vyas et al., 2018). ME supplements the MI-based motor learning process for people with intact cognitive functions (Allami et al., 2008; Ruffino et al., 2017).

Since the motor learning processes differ across individuals (Herzfeld and Shadmehr, 2014; Wu et al., 2014), significant inter-subject variability in motor behavior is anticipated that manifests in the task-specific electrical activities in the cortico-subcortical networks (Seghier and Price, 2018). Consequently, the cortical activity observed in electroencephalogram (EEG) varies across subjects during MI, impeding its utility for BCI applications (Saha et al., 2017b). A study has suggested that time-variant brain functions cause unreliable EEG signatures with poor reproducibility even within a particular subject (Meyer et al., 2013). Such inter-session, intra-subject variability together with even larger inter-subject variability confounds BCI using SMR. This review discusses how inter-session and inter-subject performance predictors could potentially augment transfer learning to improve SMR-based BCI performance while reducing calibration efforts significantly.

## 2. Sensorimotor Dynamics and BCI

### 2.1. Motor Learning Process and Brain Function

Motor variability due to variability in human kinematic parameters, e.g., force field adaptation, speed and trajectory, and motivational factors such as level of user engagement, arousal and feelings of competence, necessary for performing a motor task is an integral part of the motor learning process (Duarte and Reinkensmeyer, 2015; Úbeda et al., 2015; Edelman et al., 2019; Faller et al., 2019). Such variability does not necessarily represent noise contents only, but may potentially be a manifestation of motor and perceptual learning processes. Motor variability may augment reinforcement-based motor learning (Herzfeld and Shadmehr, 2014; Wu et al., 2014; Singh et al., 2016). Individuals with higher motor variability may learn a skill faster than individuals with lower motor variability (Wu et al., 2014; Singh et al., 2016). The EEG patterns associated with motor variability could therefore partly explain intra-individual variability in SMR-based BCI (Bradberry et al., 2010; Úbeda et al., 2015; Ostry and Gribble, 2016). Furthermore, structural and functional differences between subjects are associated with motor learning process, which might explain the motor learning variability (Tomassini et al., 2011). On the other hand, motor variability could be leveraged to augment the motor learning and rehabilitation (Krakauer, 2006; Singh et al., 2016). A study has demonstrated that alterations in EEG signatures due to motor training are dependent on intra- and inter-subject variability (Jochumsen et al., 2017).

### 2.2. Motor Imagery vs. Motor Execution

Motor imagery is the kinesthetic anticipation of corresponding overt ME without producing an actual motor output. Jeannerod stated that MI is functionally equivalent to its ME counterpart (Jeannerod, 1995). More specifically, MI is related to the preparation of ME and represents meaningful neurophysiological dynamics of human motor functions (Zich et al., 2015). Consequently, both MI and ME share common sensorimotor areas such as primary motor area (M1), supplementary motor area (SMA) and premotor cortex (PMC) (Jeannerod, 1995; Lotze and Halsband, 2006; Zich et al., 2015).

The neurophysiology underlying MI may differ in healthy people and patients with motor-impairing conditions (Lotze et al., 2001). MI-based BCI may augment the motor learning process in healthy subjects (Ruffino et al., 2017). In patients with impaired motor functions, MI is often the only viable option to drive rehabilitative BCI due to users' inability to perform overt ME (Jackson et al., 2001; Lotze and Halsband, 2006). The individuality and severity of motor impairments impact the underlying neurophysiology, for example, post-stroke neurophysiology relies on the lesion locations (Niazi et al., 2013). Studies are essential to further delineate the roles of MI and ME in motor learning or relearning for both healthy and impaired subjects to refine the design of BCI for supplementing the motor learning process.

### 2.3. Neuroplasticity and BCI-Driven Motor Rehabilitation

Rehabilitative BCI designs either attach neural prostheses to the impaired upper/lower limb or restimulate the damaged synaptic networks. In either case, the idea is to exploit and promote neural plasticity (Dobkin, 2007; Wang et al., 2010). The plastic characteristics of the brain are created by the time-variant behavior of the synapses within complex neural networks, first illustrated by Hebb, 1949 (Brown and Milner, 2003). The motor learning process and associated variability promote plasticity in the sensorimotor networks and adjust both motor and perceptual skills (Ostry and Gribble, 2016). This inherent plasticity is exploited by BCI systems to rehabilitate impaired motor functions (Dobkin, 2007). Ruffino et al. demonstrated that MI-based mental training can contribute to corticospinal plasticity (Ruffino et al., 2017). This might lead to BCI-driven rehabilitation systems for stroke and spinal cord injury patients (Niazi et al., 2013; Müller-Putz et al., 2014). Recent studies showed that BCI skill acquisition and associated physiological changes may improve BCI performance in both patients and healthy users (Perdikis et al., 2018; Edelman et al., 2019). Complex or cognitively entertaining tasks that require greater user engagement or motivation can compensate for intra- and inter-subject variability, leading to enhanced BCI learning in adverse operating conditions (Perdikis et al., 2018; Edelman et al., 2019; Faller et al., 2019; Li et al., 2019).

BCI-driven prostheses can extend the degree of freedom of users with motor impairments. The success of BCI control and rehabilitation depends on the user's capacity to modulate the intact neural ensembles (Dobkin, 2007). Substantial changes in neural substrates that were observed following closed-loop BCI-driven motor learning of prosthesis control provide evidence of neuroplasticity (Orsborn et al., 2014). In stroke patients, post-rehabilitation electromyographic recordings showed increased activity in the paretic finger following BCI-driven rehabilitation using an orthosis, which exhibits improvement in neuromuscular coherence for movement control (Ramos-Murguialday et al., 2013). Furthermore, BCI-driven proprioceptive feedback-based and functional electrical stimulation-based rehabilitation strategies could reinforce motor control (Zhao et al., 2016; Darvishi et al., 2017; Selfslagh et al., 2019).

The structural and functional changes in neural substrates induced by MI-based training with transcranial direct current stimulation or transcranial magnetic stimulation provide further evidence for the induction of neuroplasticity that is essential for motor recovery (Hong et al., 2017; Johnson et al., 2018). Because the induction of plasticity by rehabilitation varies across subjects (Leamy et al., 2014; Vallence et al., 2015), subject-specific training sessions may be required. Since the neurophysiology associated with SMR dynamics varies between individuals, quantification of variability in healthy user groups could be beneficial first step that may guide the interpretation of altered neurophysiology in diverse conditions of motor-impairment (Müller-Putz et al., 2014).

## 3. Brain Topography and BCI Performance Predictors

### 3.1. Intra- and Inter-subject Variability in Brain Topography

The functional relevance of brain topographical variability with the anatomical boundaries is still not fully understood; however, significant structure-function correspondences may be derived at the aggregate level (Honey et al., 2009, 2010). Smith et al. delineated structural differences, suggesting that the number of folds and thickness of the cortex could be associated with whole-brain functional networks (Smith et al., 2019). Furthermore, inter-subject variability in topography occurs due to subject-specific cognitive style and strategy to perform a task over time (Seghier and Price, 2018), which could augment the underlying learning processes, e.g., motor and perceptual learning (Krakauer, 2006; Baldassarre et al., 2012; Herzfeld and Shadmehr, 2014; Wu et al., 2014; Singh et al., 2016).

Intra- and inter-subject variability can be explained by scale-dependent brain networks in spatial, temporal and topological domains (Betzel and Bassett, 2017; Betzel et al., 2019). For example, diversity in spatial organization of the brain networks can be investigated either at cellular or system level. The sources of intra- and inter-subject variability in brain dynamics may be identifiable using multi-scale analysis tools (Betzel et al., 2019) although the interpretation of brain connectivity networks at different scales may not be straightforward (Raichle, 2009).

Integrating intrinsic brain activities (i.e., resting state activities) into BCI design could offer experimental and methodological advantages for scrutinizing task-specific brain dynamics (Northoff et al., 2010). While it has been argued that the brain is primarily reflexive, responding according to external stimuli/environmental demand, the brain also performs many intrinsic functions including signal acquisition, maintenance, and interpretation (Raichle, 2009, 2010). Supporting the critical role of intrinsic brain activity, it consumes 20% of the body's energy (Clarke, 1999). Thus, understanding the role of resting EEG might supplement BCI performance (Northoff et al., 2010; Suk et al., 2014; Morioka et al., 2015).

### 3.2. BCI Performance Predictors

Around 15–30% users are inherently unable to produce task-specific signature robust enough to control a BCI (Blankertz et al., 2009; Vidaurre and Blankertz, 2010). The underlying causes of this BCI illiteracy are not well-understood; however, diverse psychological and neurophysiological predictors appear to be associated with BCI performance (Blankertz et al., 2009; Vidaurre and Blankertz, 2010; Jensen et al., 2011; Hammer et al., 2012; Ahn and Jun, 2015; Jeunet et al., 2015; Reichert et al., 2015; Zhang et al., 2015; Acqualagna et al., 2016; Vasilyev et al., 2017; Sannelli et al., 2019).

Cognitive and neurological factors including functions and anatomy along with emotional and mental processes give rise to intra- and inter-subject variability affecting the performance of SMR-based BCI (Wens et al., 2014; Reichert et al., 2015; Zhang et al., 2015; Acqualagna et al., 2016; Betzel and Bassett, 2017; Vasilyev et al., 2017; Seghier and Price, 2018; Betzel et al., 2019; Smith et al., 2019). Time-variant cognitive factors such as fatigue, memory load, attention and reaction time modulate instantaneous brain activity, and can cause inconsistent SMR-based BCI performance (Hammer et al., 2012; Ahn and Jun, 2015; Fox et al., 2015; Jeunet et al., 2015; Darvishi et al., 2018; Sannelli et al., 2019). Furthermore, users' characteristics such as lifestyle, gender, and age can influence BCI performance (Ahn and Jun, 2015). Kasahara et al. illustrated that a neuroanatomical feature i.e., gray matter volume is associated with SMR-based BCI performance (Kasahara et al., 2015).

The structural and functional differences may characterize dynamic baseline activities manifested in resting-state network (RSN) dynamics. RSNs represent large-scale spatiotemporal structures exhibiting intrinsic brain activities that are thought to be functionally relevant (Deco et al., 2011). Studies have shown intra- and inter-subject variability in sensorimotor RSN, which may have implications for BCI performance variability (Jensen et al., 2011; Wens et al., 2014; Reichert et al., 2015; Zhang et al., 2015; Acqualagna et al., 2016; Vasilyev et al., 2017). It has been hypothesized that SMR-based BCI performance predictor is reliable for people who display strong resting EEG amplitudes (Blankertz et al., 2010; Suk et al., 2014; Sannelli et al., 2019). Table 1 shows a list of intra- and inter-subject BCI performance predictors.

**Table 1:** Intra- and inter-subject BCI performance predictors.

**Study**	Subject^*^	**Task type**	**Task description**	**Predictor**
Edelman et al. (2019)	68	MI, Rest	LH, RH, LH+RH	User engagement
			(Continuous cursor or	
			robotic arm control)	
Faller et al. (2019)	40	Visuo-motor	Virtual reality-based	Arousal
			plane control	
Sannelli et al. (2019)	80	MO, ME, MI	MO: LH, RH, Foot	Tiredness, imagination
			ME: LH, RH, RF	strength, motivation,
			MI: LH, RH, RF	uneasiness
Saha et al. (2019)	5	MI	RH, RF	Cortical regions
				of interest
Perdikis et al. (2018)	2 (SCI)	MI		Mutual learning
				(parameters derived
			LH, RH, LH+RH,	from interface-
			LF+RF, Rest	application, BCI output,
				and EEG)
Darvishi et al. (2018)	10	MI	LH, RH	Reaction time
				
Jochumsen et al. (2017)	47	ME	Palmar grasp	Motor training
				(laparoscopic surgery training using a simulator)
				
				
Saha et al. (2017a)	5	MI	RH, RF	Optimal Channels
				
Saha et al. (2017b)	9	MI		
			LH, RH, LF+RF,	
			Tongue	
				
Úbeda et al. (2015)	5	ME		
			Continuous Cursor	Kinematic parameters,
			control	i.e., speed, trajectory
Jeunet et al. (2015)	18			Personality and
			Motor: LH	Cognitive Profile;
		Mental	Non-motor: mental	Neurophysiological
		Imagery	rotation and	markers, including
			mental subtraction	parietal θ-power
				and frontal and
				occipital α-power
Kasahara et al. (2015)	30	MI		
			LH, RH (Finger-	Gray matter
			thumb opposition)	volume
				
Morioka et al. (2015)	51	Visuo-spatial	Attend-left	Resting EEG
		attention	or	
		task	Attend-right	
Suk et al. (2014)	83	Attention	LH, RH,	
		task	Foot	
Hammer et al. (2012)	83	MI		Visuo-motor
			LH, RH,	coordination,
			RF	ability to concentrate

* *Subjects were healthy unless specified otherwise; SCI, spinal cord injury; MI, motor imagery; ME, motor execution; MO, motor observation; LH, left hand, RH, right hand; LF, Left Foot; RF, right foot*.

## 4. Transfer Learning

### 4.1. Covariate Shift and Transfer Learning

**Figure 1:**
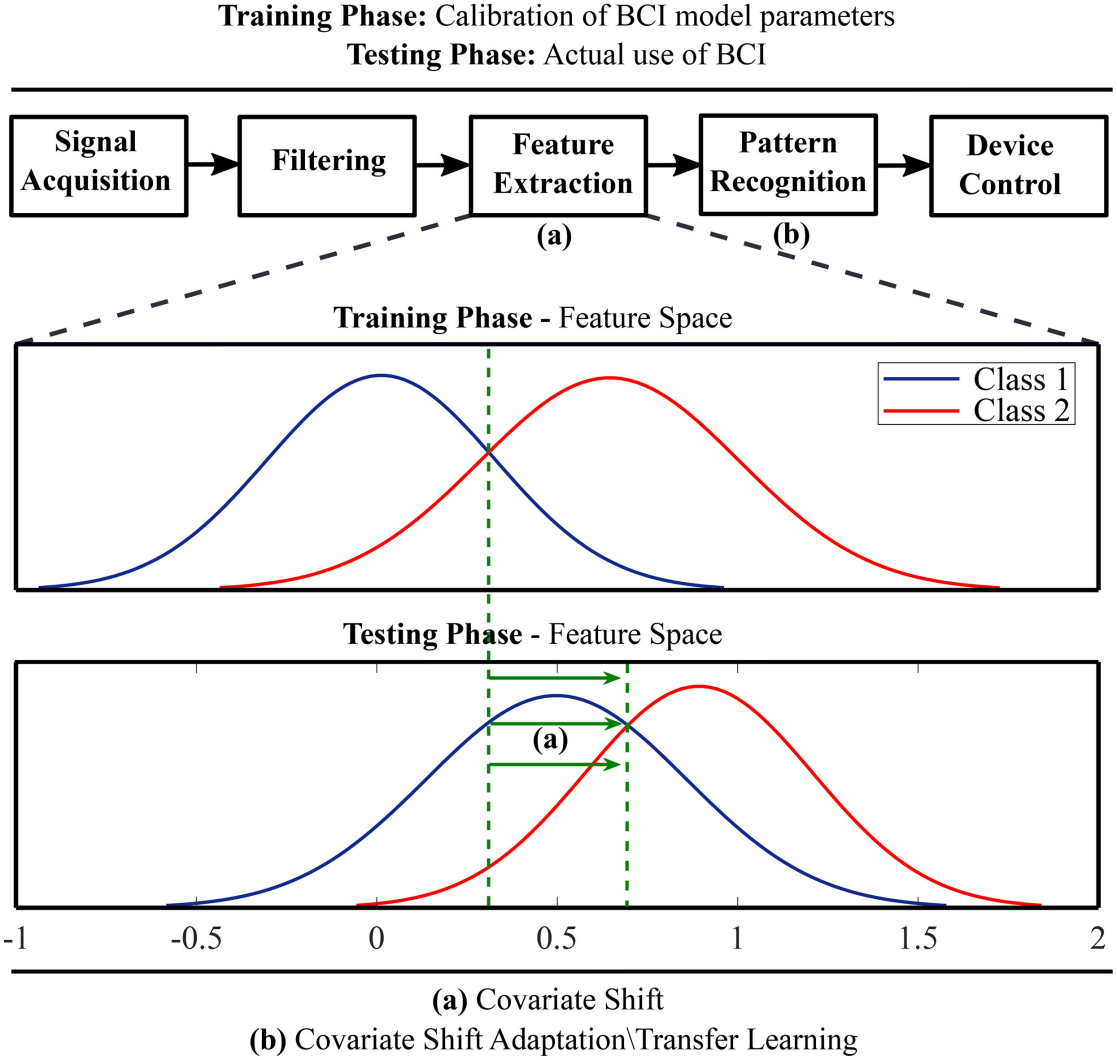
A schematic illustration of covariate shift in the feature space and application of transfer learning methods for covariate shift adaptation.

Transfer learning techniques originating from the field of machine learning have been adopted to compensate BCI systems for inter-subject and inter-session variability of EEG feature distributions (Fazli et al., 2015; Jayaram et al., 2016). A key idea is to regularize BCI model parameters for covariate shift adaptation. Covariate shift occurs when distributions of training and test data differ significantly although their conditional distributions may remain unchanged (Krusienski et al., 2011). Figure 1 schematically illustrates the idea of covariate shift when the training and test data distributions are different. The underlying time-variant and subject-specific brain dynamics depends on associated psychological and neurophysiological factors (Blankertz et al., 2009; Vidaurre and Blankertz, 2010; Jensen et al., 2011; Hammer et al., 2012; Ahn and Jun, 2015; Jeunet et al., 2015; Reichert et al., 2015; Zhang et al., 2015; Acqualagna et al., 2016; Vasilyev et al., 2017; Sannelli et al., 2019) and cause covariate shift in EEG-derived feature distributions (Krusienski et al., 2011; Fazli et al., 2015; Jayaram et al., 2016).

The earliest attempts to overcome inter-session variability include preliminary training sessions to enhance the user's ability to modulate brain signals robust enough to control BCI (Wolpaw et al., 1991; Wolpaw and McFarland, 1994; Birbaumer et al., 1999). The training sessions required for users are tedious and inconvenient. Therefore, machine learning-based BCI models were introduced to reduce individual training session for each BCI use, in which a model has to be calibrated based on the data at the beginning of each session (Ramoser et al., 2000; Blankertz et al., 2002). Recent studies have proposed SMR-based BCI without any session- and subject-specific calibration utilizing the concept of transfer learning (Kang et al., 2009; Li et al., 2010; Lu et al., 2010; Niazi et al., 2013; Kang and Choi, 2014; Fazli et al., 2015; Lotte, 2015; Jayaram et al., 2016; Saha et al., 2017a,b, 2019; Fahimi et al., 2018; He and Wu, 2019).

### 4.2. The Concept of Inter-subject Associativity

Most of the existing transfer learning approaches are based on regularization or inter-session/subject transfer of model parameters, indirectly transferring knowledge pertaining to the sources of intra- and inter-subject variability (Samek et al., 2013; Lotte, 2015). Many works on transfer learning for SMR-based BCI proposed the use of a very few training samples from the target subject (Kang et al., 2009; Lu et al., 2010; Kang and Choi, 2014; Fahimi et al., 2018; He and Wu, 2019). Recent studies have utilized resting EEG from the target subject incorporated into transfer learning model before proceeding to the actual experiment (Suk et al., 2014; Morioka et al., 2015). While time and effort for building those models could be significantly reduced, they still require training session. Others have recently demonstrated the feasibility of inter-subject BCI models without any training trial from the target subject (Saha et al., 2017a,b, 2019). However, the performance requires to be improved significantly prior to real-life use of such BCI systems.

A transfer learning method is worthwhile if the subjects share non-stationarities that can be modeled in an inter-subject context, but ineffective if the subjects exhibit unlike non-stationarities (Samek et al., 2013). The term *inter-subject associativity* refers to potential inter-subject BCI performance predictors, which could be incorporated into BCI design to augment transfer learning (Kang and Choi, 2014; Wronkiewicz et al., 2015; Saha et al., 2017a,b, 2019). Source-space analysis for detecting inter-subject associative EEG channels can improve SMR-based BCI performance (Wronkiewicz et al., 2015; Saha et al., 2017a, 2019). For example, the classification accuracies for two different subject pairs are 90.36±5.59% and 63.21±8.43%, respectively, suggesting not both subject pairs can be used to achieve a good performance (Saha et al., 2019).

A set of generalized BCI frameworks would be more feasible to implement as compared to a common BCI framework for all users. Because, it is evident to observe significant inter-subject variability in EEG signals (Saha et al., 2017b). Successful quantification of *inter-subject associativity* may suggest clustering of subjects, each cluster having subjects with EEG signal characteristics that are similar or can be interpreted in an inter-subject context. Considering the increasing volume of EEG-BCI databases, it may become feasible to quantify the exact sources of inter-subject/session variability as well as indicators of inter-subject associativity allowing to reduce training sessions to a minimum (Lotte, 2015). Recent advances in deep learning methods demonstrate a potential application that alleviates intra- and inter-subject variability in BCI settings (Chiarelli et al., 2018; Fahimi et al., 2018). Meanwhile, recent studies suggest that the quantification of *inter-subject associativity* could be equally important to increase the efficacy of exclusively machine learning-based transfer learning strategies for covariate shift adaptation (Kang et al., 2009; Kang and Choi, 2014; Wronkiewicz et al., 2015; Saha et al., 2017b, 2019; Perdikis et al., 2018).

## 5. Conclusion

Intra- and inter-subject variability is undeniable due to time-variant factors related to the experimental setting and underlying psychological and neurophysiological parameters. Besides the extensive use of transfer learning methods for the covariate shift adaptation, many recent works sought to find suitable psychological and neurological predictors for BCI performance. The assimilation of such predictors into a subject independent context may reduce or eliminate the tedious session or subject-specific training by supplementing the performance of existing transfer learning methods. However, collecting *a priori* information related to BCI performance predictors could be challenging. Inter-subject topographical associativity characterized by resting EEG could provide a viable alternative solution to reduce the calibration time to a minimum (Northoff et al., 2010; Suk et al., 2014; Morioka et al., 2015) assuming we understand the significance of intrinsic brain activities, i.e., resting EEG signals, and the role of RSN topographies on SMR-related brain functions.

## Author Contributions

SS conceived the idea, prepared the figure and table, and wrote the first draft. MB reviewed and commented on the manuscript. SS and MB read and approved the final manuscript.

### Conflict of Interest

The authors declare that the research was conducted in the absence of any commercial or financial relationships that could be construed as a potential conflict of interest.
